# Magnetic Resonance Findings of the Corpus Callosum in Canine and Feline Lysosomal Storage Diseases

**DOI:** 10.1371/journal.pone.0083455

**Published:** 2013-12-27

**Authors:** Daisuke Hasegawa, Shinji Tamura, Yuya Nakamoto, Naoaki Matsuki, Kimimasa Takahashi, Michio Fujita, Kazuyuki Uchida, Osamu Yamato

**Affiliations:** 1 Division of Veterinary Radiology, Department of Veterinary Science, Nippon Veterinary and Life Science University, Tokyo, Japan; 2 Tamura Animal Clinic, Hiroshima, Japan; 3 Kyoto Animal Referral Medical Center, Kyoto, Japan; 4 Department of Veterinary Clinical Pathobiology, Graduate School of Agricultural and Life Science, The University of Tokyo, Tokyo, Japan; 5 Division of Veterinary Pathology, Department of Veterinary Science, Nippon Veterinary and Life Science University, Tokyo, Japan; 6 Department of Veterinary Pathology, Graduate School of Agricultural and Life Science, The University of Tokyo, Tokyo, Japan; 7 Laboratory of Clinical Pathology, Department of Veterinary Medicine, Kagoshima University, Kagoshima, Japan; Van Andel Institute, United States of America

## Abstract

Several reports have described magnetic resonance (MR) findings in canine and feline lysosomal storage diseases such as gangliosidoses and neuronal ceroid lipofuscinosis. Although most of those studies described the signal intensities of white matter in the cerebrum, findings of the corpus callosum were not described in detail. A retrospective study was conducted on MR findings of the corpus callosum as well as the rostral commissure and the fornix in 18 cases of canine and feline lysosomal storage diseases. This included 6 Shiba Inu dogs and 2 domestic shorthair cats with GM1 gangliosidosis; 2 domestic shorthair cats, 2 familial toy poodles, and a golden retriever with GM2 gangliosidosis; and 2 border collies and 3 chihuahuas with neuronal ceroid lipofuscinoses, to determine whether changes of the corpus callosum is an imaging indicator of those diseases. The corpus callosum and the rostral commissure were difficult to recognize in all cases of juvenile-onset gangliosidoses (GM1 gangliosidosis in Shiba Inu dogs and domestic shorthair cats and GM2 gangliosidosis in domestic shorthair cats) and GM2 gangliosidosis in toy poodles with late juvenile-onset. In contrast, the corpus callosum and the rostral commissure were confirmed in cases of GM2 gangliosidosis in a golden retriever and canine neuronal ceroid lipofuscinoses with late juvenile- to early adult-onset, but were extremely thin. Abnormal findings of the corpus callosum on midline sagittal images may be a useful imaging indicator for suspecting lysosomal storage diseases, especially hypoplasia (underdevelopment) of the corpus callosum in juvenile-onset gangliosidoses.

## Introduction

Lysosomal storage diseases are congenital, inherited neurodegenerative diseases observed in dogs and cats as well as in humans. Several previous studies reported clinical, biochemical, pathologic, and imaging features of lysosomal storage diseases [1]–[9]. In particular, juvenile-onset lysosomal storage diseases, such as GM1 and GM2 gangliosidoses, show abnormalities of signal intensities in the subcortical white matter of the cerebrum on magnetic resonance imaging (MRI), which may be caused by hypomyelination and/or dysmyelination [4], [6], [8]. GM1 gangliosidosis in Shiba Inu dogs [8] and GM2 gangliosidosis variant 0 in Japanese domestic shorthair cats [4] and in familial toy poodles [6] show diffuse and symmetrical hyperintensity on T2-weighted images in the subcortical white matter of the whole cerebrum from birth to death.

**Figure 1:**
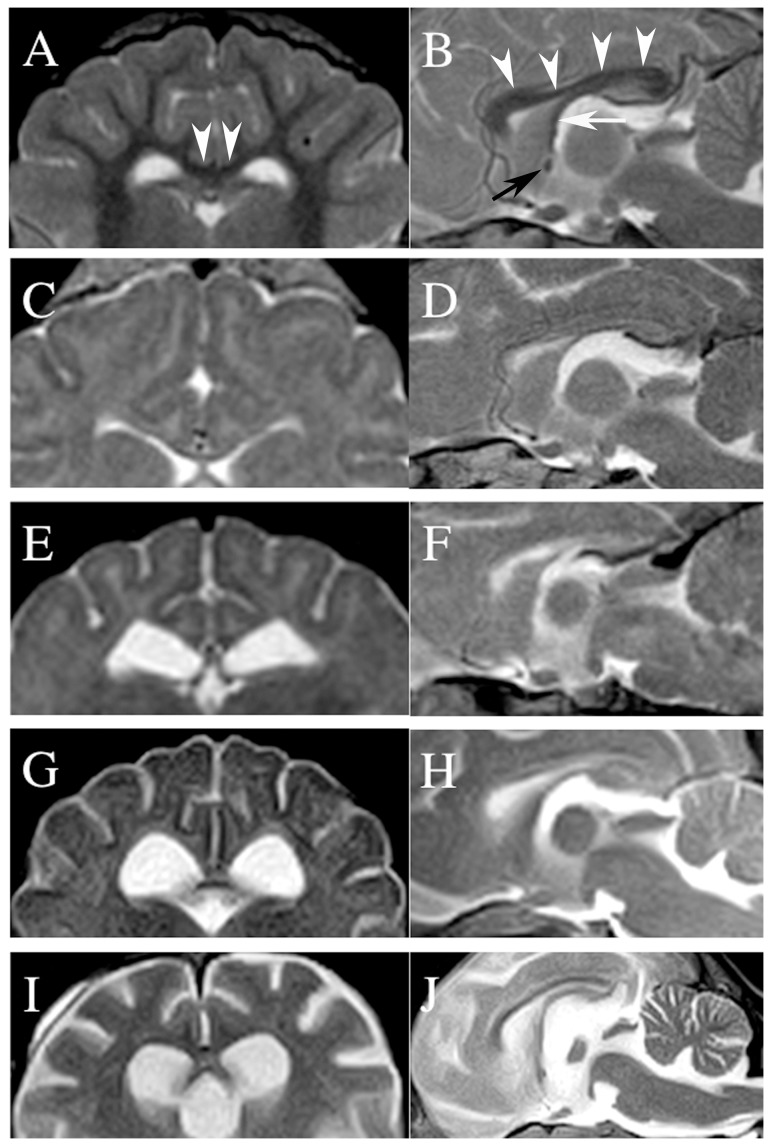
Transverse (A, C, E, G, I) and midline sagittal (B, D, F, H, I) T2-weighted MR images of the corpus callosum. A and B: a 4-month-old normal Beagle dog. Arrowheads (A, B) indicate the corpus callosum, the black arrow (B) indicates the rostral commissure, and the white arrow (B) indicates the fornix. C and D: a 6-month-old Shiba Inu dog with GM1 gangliosidosis (Case no. 1 in Table 1). E and F: a 7-month-old domestic shorthair cat with GM2 gangliosidosis (Case no. 9). G and H: a 21-month-old toy poodle with GM2 gangliosidosis (Case no. 11). I and J: a 19-month-old chihuahua with neuronal ceroid lipofuscinosis (Case no. 18). For detailed evaluations of the corpus callosum, rostral commissure, and fornix in each case refer to Table 1.

On the other hand, the corpus callosum is the largest tract of commissural fibers connecting both sides of the cerebral hemisphere. If normal, this structure is easy to recognize in MRIs of the midline sagittal plane as an eyebrow-like bold line overlying the fornix and third ventricle (Figures 1A, B). Previous imaging studies of lysosomal storage diseases focused on the subcortical white matter, but did not describe the corpus callosum. Consequently, image findings of the corpus callosum in canine and feline lysosomal storage diseases may have been overlooked. Most recently, one study evaluated corpus callosal volume as a therapeutic marker of canine mucopolysaccharidosis type I [10]; however, they used middle (1.5 Tesla (T)) to high (3.0 T) magnetic fields and mucopolysaccharidosis type I colony dogs, and did not focus on clinical diagnosis. Therefore, we retrospectively investigated MR images of the corpus callosum in canine and feline gangliosidoses and canine neuronal ceroid lipofuscinosis cases. We also focused on the fornix and rostral commissure, structures around the corpus callosum. The objective of this study was to examine the MR appearance of the corpus callosum in some lysosomal storage diseases and to evaluate whether these findings may be useful as imaging indicators for canine and feline lysosomal storage diseases.

## Materials and Methods

### Ethics statement

This was a retrospective, multi-institutional study using the diagnostic MRI data that were obtained from animal patients in some private and university animal hospitals. The medical information and data of the animals were obtained with the informed consent of their owners. All procedures using data and specimens were performed in accordance with guidelines regulating animal use and ethics at the Animal Medical Center of Animal Medical Center of Nippon Veterinary and Life Science University.

### Cases and data collection

The cases studied are summarized in Table 1. Medical and MRI data were collected from four institutes and included the following: 6 Shiba Inu dogs (including previously reported cases [8]) and 2 domestic shorthair cats with GM1 gangliosidosis, 2 domestic shorthair cats (including previously reported cases [4]), 2 familial toy poodles [6], and a golden retriever [3] with GM2 gangliosidosis variant 0 (Sandhoff disease), and 2 border collies (including a previously reported case [1]) and 3 chihuahuas [7] with neuronal ceroid lipofuscinosis were included. All affected animals were diagnosed definitively by pedigree analysis, genetic tests, lysosomal enzyme activities, thin-layer chromatographs, or histopathology (see individual references and Table 1).

**Table 1:** Summary of Studied Cases and Those MR Findings.

								MR findings						
No.	Type of LSD	Breed/Sex	Age of clinical onset (mo)	Onset type	Age of 1^st^ MRI (mo)	Magnet strength (T)	ST (mm)	CC	RC	Fx	Method of definitive diagnosis	Gene	Mutation	Pathology
1	CGM1	Shiba/M	5	J	2	1.5	2.5	PV	VA	VA	G, LE, TLC, P	*GLB1*	c.1647delC	D
2	CGM1	Shiba/M	5	J	3	0.3	3.0	PV	NV	VA	G	*GLB1*	c.1647delC	NE
3	CGM1	Shiba/F	5	J	6	0.3	4.0	PV	NV	PV	G	*GLB1*	c.1647delC	D
4	CGM1	Shiba/F	6	J	8	0.4	4.0	PV	VA	PV	G	*GLB1*	c.1647delC	NE
5	CGM1	Shiba/F	6	J	7	0.3	3.0	PV	NV	VA	G, P	*GLB1*	c.1647delC	D
6	CGM1	Shiba/M	6	J	7	0.3	3.5	PV	VA	VA	G	*GLB1*	c.1647delC	NE
7	FGM1	DSH/M	4	J	7	0.3	2.5	PV	VA	VA	G	*GLB1*	c.1448G>C	NE
8	FGM1	DSH/F	6	J	9	0.3	3.0	PV	VA	PV	G	*GLB1*	c.1448G>C	NE
9	FGM2	DSH/M	2	J	7	1.5	2.5	NV	NV	PV	G, LE, TLC, P	*HEXB*	c.667C>T	D
10	FGM2	DSH/F	3	J	3	0.4	3.0	PV*	NV*	PV*	G, LE	*HEXB*	c.667C>T	D
11	CGM2	TP/F	12	LJ	13	0.3	4.0	PV	NV	VA	G, P	*HEXB*	c.283delG	D
12	CGM2	TP/F	11	LJ	19	0.3	4.0	PV*	NV*	PV*	G, P	*HEXB*	c.283delG	D
13	CGM2	GR/M	11	LJ	11	0.3	4.0	VA*	VA*	VA*	LE, TLC	*HEXB*	ND	NE
14	NCL	BC/F	18	EA	24	0.3	3.0	VA	VA	VA	G	*CLN5*	c.619C>T	NE
15	NCL	BC/M	16	EA	24	0.3	6.0	VA	VA	PV	G	*CLN5*	c.619C>T	D
16	NCL	CH/M	18	EA	21	0.3	3.0	VA	VA	VA	P	ND	ND	D
17	NCL	CH/F	16	EA	18	0.3	4.0	VA	VA	PV	PA	ND	ND	NE
18	NCL	CH/M	06	EA	19	0.3	4.0	VA	VA	VA	P	ND	ND	D

The results of the classification of MR findings in this table indicate the majority decision of the three reviewers.

LSD, lysosomal storage disease; mo, months; T, Tesla; ST, slice thickness; CC, corpus callosum; RC, rostral commissure; CGM1, canine GM1 gangliosidosis; FGM1, feline GM1 gangliosidosis; CGM2, canine GM2 gangliosidosis; FGM2, feline GM2 gangliosidosis; NCL, neural ceroid lipofuscinosis; DSH, domestic shorthair; TP, toy poodle; GR, golden retriever; BC, border collie; CH, chihuahua; M, male; F, female; J, juvenile-onset; LJ, late juvenile-onset; EA, early adult-onset; N, normal; VA, visualized but atrophic; PV, partially visualized; NV, not visualized; *, lack of sagittal plane; G, gene test; LE, leukocyte enzyme activities; TLC, thin-layer chromatography; P, pathology; PA, pedigree analysis; ND, not determined; D, done; NE, not examined.

Because this study was a retrospective review of imaging findings, describing the clinical parameters of each case was beyond the object of this study. These were described in detail in each of the previous reports. The clinical features of each type of lysosomal storage disease are summarized below.

#### GM1 gangliosidosis in Shiba Inu dogs

Clinical signs including cerebellar ataxia (hypermetric) began at 5–6 months and worsened progressively, with cerebral signs appearing at 7–8 months, atactic abasia or astasia at 9–10 months, and lethargy to death at approximately 11 months. This disease is an autosomal recessive deficiency of β-galactosidase enzyme activity due to mutation of the *GLB1* gene. Detailed clinical, imaging, histological, and molecular features have been reported previously [8].

#### GM1 gangliosidosis in domestic shorthair cats

Clinical signs, including cerebellar ataxia (hypermetric) and head tremor, began at 4–6 months and worsened progressively, with atactic abasia or astasia appearing at 7–9 months, and lethargy to death at approximately 11–12 months (unpublished). This disease is an autosomal recessive deficiency of β-galactosidase enzyme activity due to mutation of the *GLB1* gene [11].

#### GM2 gangliosidosis in domestic shorthair cats

Cerebellar signs were described starting from 2–3 months. Neurological signs, including mental status, postural reactions, and spinal reflexes, deteriorated progressively, and the animals became critical and died at 7–10 months. Cases in this study had a mutation in the *HEXB* gene encoding β-hexosaminidase. Detailed information was reported previously [4], [5].

#### GM2 gangliosidosis in familial toy poodles

Clinical signs, including motor disorders and tremor, began at approximately 9–12 months. The animals died of neurological deterioration at 18–23 months. This disease is an autosomal recessive deficiency of β-hexosaminidase A and B enzyme activites due to a frameshift mutation of the *HEXB* gene. Detailed clinical, imaging, histological, and molecular features have been reported previously [6], [12].

#### GM2 gangliosidosis in a golden retriever

Neurologic abnormalities, including depression, dementia, ataxia, and blindness, were observed from 11 months, progressed into stupor at 15 months, and the dog died one week later. As the owner did not permit a necropsy, GM2 gangliosidosis variant 0 (Sandhoff disease) was diagnosed by leukocyte enzyme activities and thin-layer chromatography, and the causative gene was not determined [3].

#### Neuronal ceroid lipofuscinosis in border collies

Clinical signs including behavioral abnormalities began at 15–20 months and worsened progressively, with motor dysfunction and visual impairments appearing at 19–23 months, and lethargy to death at 23–32 months. This disease is an inherited disease characterized by lipopigment deposition in the neurons and other cells of the body due to mutation of the ceroid-lipofuscinosis, neuronal 5 (*CLN5*) gene. Detailed clinical, imaging, histological, and molecular features were reported previously [1], [2].

#### Neuronal ceroid lipofuscinosis in chihuahuas

Clinical signs, including behavioral abnormalities and visual impairments, began at 16–18 months. The animals died of neurological deterioration at 23–24 months. This disease is an inherited disease characterized by lipopigment deposition in the neurons and other cells of the body. The gene mutation site was not detected. Detailed clinical, imaging, histological features were reported previously [7].

As described above, these cases of lysosomal storage diseases may be classified into three groups based on the age at clinical onset (Table 1): 1) the juvenile-onset group consisted of the Shiba Inu dogs (clinical onset; 5–6 months) [8] and domestic shorthair cats (5–7 months) with GM1 gangliosidosis and the domestic shorthair cats (2–3 months) [3] with GM2 gangliosidosis; 2) the late juvenile-onset group consisted of the toy poodles (9–11 months) [6] and a golden retriever (11 months) [3] with GM2 gangliosidosis; and 3) the early adult-onset group consisted of the border collies (15–20 months) and chihuahuas (16–18 months) with canine neuronal ceroid lipofuscinosis [1], [2], [7].

### MRI and evaluation

The MRI systems and obtained sequences varied among the four institutes. The field strengths of the MRIs varied from 0.3 to 1.5 T, and the slices thicknesses of the 18 cases varied from 2.5 to 6.0 mm (Table 1). In this study, serial, transverse T1- and T2-weighted images and midline sagittal T2-weighted images were reviewed as main subjects, and transverse and sagittal images of T1-weighted and fluid-attenuated inversion recovery images were also reviewed, if available. However, no sagittal images were obtained from one of the cats (Case no. 10), one of the toy poodles (Case no. 12), and the golden retriever (Case no. 13).

Visual reviews of the corpus callosum, rostral commissure, and fornix were evaluated in each case by three experienced neurologist/neuroradiologists (designated as A, B, C for DH, ST, YN, respectively) who were blinded to the condition of the animal. In reviewing the objects, they referred to the images of normal and age-matching animals as control images. The normal and age-matching animals were: an 8-months-old Shiba Inu dog (0.4T), a 4-months-old beagle (1.5T; shown in Fig. 1A, B), a 2-year-old Border Collie (1.5T) and a 5-months-old normal cat (1.5T). Evaluations of the MR findings of each structure were classified as ‘normal’, ‘visualized but atrophic’, ‘partially visualized’, and ‘not visualized’. ‘Normal’ indicates that the structure was visualized clearly and consistently and had sufficient thickness and signal intensity similar to those of normal animals, as shown in Figures 1A and B. ‘Visualized but atrophic’ indicates that the structure was visualized consistently but was significantly thinner or showed decreased signal intensity on all reviewed planes (transverse and sagittal images). ‘Partially visualized’ indicates that the structure was visualized but was partially undetectable or was visualized only intermittently. ‘Not visualized’ indicates that the structure could not be confirmed on all reviewed planes. The agreement in the evaluations by the three reviewers was analyzed by weighted kappa (κ) statistics.

## Results

The results of the MR findings, which were decided by majority of three reviewers, of the corpus callosum, rostral commissure, and fornix of all 18 cases are summarized in Table 1. The agreements between the reviewers for each structure were excellent, and are shown in Table 2 (κ coefficient 0.80 <), with those of corpus callosum showing the highest (κ = 0.95) and those of rostral commissure the lowest (κ = 0.81).

**Table 2:** The agreement between reviewers by weighted κ statistics.

	A∶B	A∶C	B∶C	mean
CC	0.98	0.93	0.93	0.95
RC	0.90	0.78	0.76	0.81
Fx	0.94	0.94	0.95	0.94

Values show κ coefficients. A, B and C refers to each reviewer. CC, corpus callosum; RC, rostral commissure; Fx, fornix.

The corpus callosum was ‘not visualized’ or ‘partially visualized’ in all cases of the juvenile-onset group, including the Shiba Inu dogs. It was barely recognized on both the transverse and sagittal images (Figures 1C, D), and in the domestic shorthair cats with GM1 gangliosidosis (data not shown) and GM2 gangliosidosis (Figures 1E, F). In the late juvenile-onset group, visualization of the corpus callosum in the toy poodles (Figures 1G, H) and golden retriever (data not shown) with canine GM2 gangliosidoses was inconsistent (‘partially visualized’ or ‘visualized but atrophic’), although the sagittal plane was not obtained from 2 of the 3 cases. In those 2 groups, if the corpus callosum was evaluated as ‘partially visualized’, the visualized portion was significantly atrophic (Figure 1C) and shortened longitudinally (Figure 1H). On the other hand, the corpus callosum of the early adult-onset group, comprised of those with canine neuronal ceroid lipofuscinosis, was recognized clearly and consistently, although it was thinner than those of normal animals (Figures 1I, J).

The rostral commissure was not visualized in 5 of 10 cases in the juvenile-onset group (Figures 1F) or in 2 of 3 cases in the late juvenile-onset group (Figure 1H). Similar to the corpus callosum, the rostral commissures of the early-adult group were all visualized, but atrophic (Figure 1J). On the other hand, the fornix was recognized in all cases in the present study, but the degree of recognition varied among the cases.

**Figure 2:**
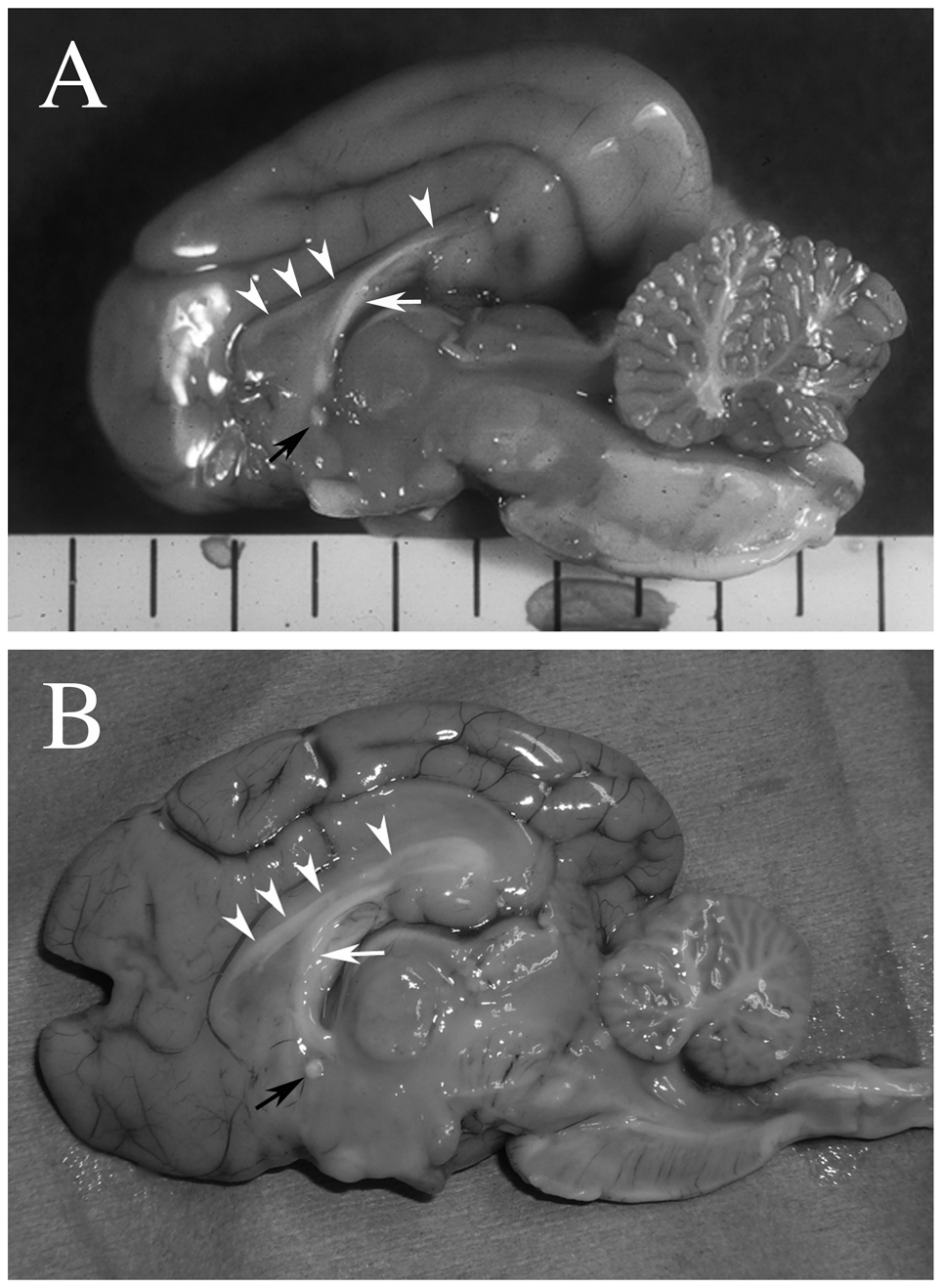
Macroscopic photographs of the midline sagittal section from a domestic shorthair cat (A: corresponding to Figure 1F) and a toy poodle (B: corresponding to Figure 1H) with GM2 gangliosidosis. The corpus callosum (arrowheads, although very pale in A), the rostral commissure (black arrow), and the fornix (white arrow) are grossly confirmed in both cases.

In all cases that underwent necropsy and histopathological investigation (2 dogs with GM1 gangliosidosis, 2 cats and the 2 toy poodles with GM2 gangliosidosis, and 2 dogs with neuronal ceroid lipofuscinosis), the corpus callosum and the rostral commissure were observed both macroscopically and microscopically, although they were extremely thin and poorly myelinated (dysmyelination) (Figure 2). In contrast, these structures were relatively well developed in the other necropsied neuronal ceroid lipofuscinosis cases (1 chihuahua and 1 border collie).

## Discussion

In the present study, imaging findings of the corpus callosum, rostral commissure, and fornix in cases of canine and feline lysosomal storage diseases were described for veterinary researchers and/or practitioners. Our intent was not to investigate the pathophysiological mechanisms of lysosomal storage diseases, including pathological findings of subcortical white matter in detail, because those have been discussed previously [1]–[9].

The corpus callosum is the largest commissural and myelinated fiber tract connecting the cerebral cortices. In dogs, the corpus callosum is recognized on sagittal T2-weighted MR images at 6 weeks of age and assumes an adult appearance at 16 weeks [13]. The rostral commissure connects both sides of the rhinencephalon, and the fornix is a complex of fibers, including projection fibers from the hippocampi and commissural fibers, connecting both sides of the limbic system. Although there is no information about the development of the rostral commissure and the fornix, it is likely similar to the corpus callosum [13]. We could not find any reports that described the development of the corpus callosum using conventional (clinical) MRI in cats. However, the corpus callosum was recognized at postnatal day 35 in a diffusion tractographic study [14], and we also confirmed the corpus callosum on conventional MRI in a 2-month-old, clinically normal cat (unpublished data). In infantile-onset lysosomal storage diseases (e.g., <4 weeks old), these structures may not be sufficiently developed to be visualized. However, in the present study, all cases of lysosomal storage diseases were over 2 months old, and so we should have been able to confirm the corpus callosum, if normal.

In the juvenile-onset group, the corpus callosum was difficult to recognize on MRI, although the extremely thinned callosum was detected on necropsy (Fig. 1F and Fig. 2A). On the midline sagittal MR images, a complete visual deficit of the corpus callosum sometimes appeared (Fig. 1D, F). This pattern of “hypoplasia of the corpus callosum” may result via a similar mechanism as that for subcortical white matter, i.e. underdevelopment, as discussed in previous reports [8]. In addition, increased signal intensity of the corpus callosum, similar to that of subcortical white matter on T2-weighted images, may have been responsible for the lack of visualization on MRI. Alternatively, it is possible that the extremely thin corpus callosum with extended ventricles and/or longitudinal fissure could not be visualized due to slice angle, slice thickness, resolution, or field strength of the MRI. For example, images of the corpus callosum in the Shiba Inu dog with GM1 gangliosidosis that were obtained using a 1.5 Tesla system were slightly visible in the transverse plane, as shown in Figure 1C; however, it could not be clearly observed (almost visually deficit) in the sagittal plane, as shown in Figure 1D. This inconsistency may arise from the characteristic of the midline slice of the sagittal plane. On the midline, the corpus callosum is sandwiched between the longitudinal cerebral fissure and the third ventricle filled with cerebrospinal fluid. MR images have a slice thickness and the visualized images are the means of signal intensities within the slice thickness. Thus, on the midline sagittal plane, the signal intensity of the extremely thinned corpus callosum that is briefly in the right and left direction is masked visually by higher signal intensities of cerebrospinal fluid (partial volume effect). Because this phenomenon even occurred in 1.5 Tesla, it is all the more remarkable in the lower field that used a more thickened slice. By contrast, this is hard to visualize on the transverse plane, since the corpus callosum continues in anterior-posterior direction. Therefore, it will be possible that a very thin corpus callosum may be recognized if its images are obtained using a higher strength MRI system that can apply a thinner slice (e.g., ≥3.0 Tesla).

On the other hand, each of the early adult-onset group of neuronal ceroid lipofuscinosis cases exhibited hypointensity on T2-weighted images and a clear corpus callosum, although they were thinner than normal. In one case of this group (Case no. 15), images had been obtained at 6.0 mm of slice thickness. In spite of increased partial volume effect that was explained above, a thinner callosum was able to be detected. That is to say, the corpus callosum of this group has a certain volume compared to the juvenile-onset (hypoplasia) group. This may represent degenerative changes, i.e. atrophy, that thinned the once normally developed callosum along with the progression of parenchymal atrophy, which was confirmed on a time course MRI follow up in one of the cases of neuronal ceroid lipofuscinosis (unpublished data). Furthermore, in a report of fucosidosis in a domestic shorthair cat (adult-onset), the corpus callosum and the rostral commissure were visualized, although they were thin and less clear than normal [9]. As described in the Introduction, a recently published study evaluated corpus callosal volume as a therapeutic marker of canine mucopolysaccharidosis type I with or without enzyme replacement therapy [10]. Although mucopolysaccharidosis type I is a juvenile-onset (3–6 months) lysosomal storage disease [15], its initial clinical signs are of musculoskeletal and joint disease rather than neurological disease, and there are no published MRI findings in the juvenile stage. The MRI findings of the corpus callosum in the untreated mucopolysaccharidosis type I dogs examined at 12–28 months were similar to those of the early adult-onset group in the present study [10]. The neurological and/or cerebral status of canine mucopolysaccharidosis type I may be classified into the early adult- or adult-onset group, although not without some speculation. Nevertheless, the canine mucopolysaccharidosis type I study showed that the corpus callosum maybe a useful imaging marker [10], which supports the findings of the present study.

Compared with the findings of the 2 groups described above (juvenile- and early adult-onset), those of canine GM2 gangliosidosis (late-juvenile onset group) are inconsistent. The corpora callosa of the toy poodles with GM2 gangliosidosis were unrecognizable, and those with subcortical white matter also showed diffuse hyperintensity on T2-weighted images, similar to the juvenile-onset group. In contrast, the corpus callosum of the affected golden retriever resembled those of the early adult-onset group (canine neuronal ceroid lipofuscinosis), in that it was clearly visible on transverse images and there was no T2-hyperintensity of the subcortical white matter. This difference in imaging findings of the white matter including the corpus callosum may arise from differences in gene mutations, even though they are both GM2 gangliosidosis variant 0 (Sandhoff-like disease). In other words, based on these MRI findings, the pathogenic mutations of the disease in the toy poodles [12] and the golden retriever [3] may be different, keeping in mind that the mutation in the latter has not been identified. However, only the T2-hyperintensity spot in the caudate nuclei was common to the diseases in both the toy poodles and the golden retriever. Therefore, this finding may be a specific marker for suspecting canine GM2 gangliosidosis variant 0. Furthermore, the MRI findings in another type of GM2 gangliosidosis (B variant; Tay-Sachs disease) in 2 Japanese Chins were just reported [16]. The initial clinical signs of this type of GM2 gangliosidosis seem to begin at 11–16 months, and thus are classified as late juvenile- or early adult-onset. The corpora callosa of the Japanese Chins were clearly visualized, although they were slightly atrophic (equivalent to ‘visualized but atrophic’ in the present study).

In conclusion, although MRI findings of the corpus callosum are varied among the types of lysosomal storage diseases, hypoplasia of the corpus callosum was observed consistently in the juvenile-onset gangliosidoses. Therefore, findings of hypoplasia of the corpus callosum, especially in the midline sagittal plane, may be a useful imaging indicator for suspecting gangliosidoses. On the other hand, an extremely thin but confirmable, i.e. atrophic, corpus callosum was also observed in all cases of relatively late-onset lysosomal storage diseases. The findings of the rostral commissure and the fornix may also have some utility, but its impression on MRI will be less than that of the corpus callosum. Consequently, it is important that clinicians pay attention to imaging findings of the corpus callosum when any lysosomal storage diseases are suspected. Conversely, lysosomal storage diseases, especially gangliosidoses, should be included in the differential diagnosis of abnormal findings of the corpus callosum. In the future, if many controls and cases can be collected for each age, breed and disease, quantitative or rational analyses should be enabled of the corpus callosum as an objective marker for lysosomal storage diseases.
